# RECQ1 expression is upregulated in response to DNA damage and in a p53-dependent manner

**DOI:** 10.18632/oncotarget.18237

**Published:** 2017-05-27

**Authors:** Swetha Parvathaneni, Xing Lu, Ritu Chaudhary, Ashish Lal, Srinivasan Madhusudan, Sudha Sharma

**Affiliations:** ^1^ Department of Biochemistry and Molecular Biology, College of Medicine, Howard University, NW, Washington, DC, 20059, USA; ^2^ Regulatory RNAs and Cancer Section, Genetics Branch, National Cancer Institute, National Institutes of Health, Bethesda, MD, 20892, USA; ^3^ Academic Unit of Oncology, Division of Cancer and Stem Cells, School of Medicine, University of Nottingham, Nottingham, NG51PB, UK

**Keywords:** RecQ, helicase, DNA damage, p53, gene expression

## Abstract

Sensitivity of cancer cells to DNA damaging chemotherapeutics is determined by DNA repair processes. Consequently, cancer cells may upregulate the expression of certain DNA repair genes as a mechanism to promote chemoresistance. Here, we report that RECQ1, a breast cancer susceptibility gene that encodes the most abundant RecQ helicase in humans, is a p53-regulated gene, potentially acting as a defense against DNA damaging agents. We show that RECQ1 mRNA and protein levels are upregulated upon treatment of cancer cells with a variety of DNA damaging agents including the DNA-alkylating agent methylmethanesulfonate (MMS). The MMS-induced upregulation of RECQ1 expression is p53-dependent as it was observed in p53-proficient but not in isogenic p53-deficient cells. The RECQ1 promoter is bound by endogenous p53 and is responsive to p53 in luciferase reporter assays suggesting that RECQ1 is a direct target of p53. Treatment with the chemotherapeutic drugs temozolomide and fotemustine also increased RECQ1 mRNA levels whereas depletion of RECQ1 enhanced cellular sensitivity to these agents. These results identify a previously unrecognized p53-mediated upregulation of RECQ1 expression in response to DNA damage and implicate RECQ1 in the repair of DNA lesions including those induced by alkylating and other chemotherapeutic agents.

## INTRODUCTION

Genes encoding proteins that function in replication checkpoint, maintenance of stalled replication forks and homologous recombination repair are frequently upregulated in a variety of cancers [1–3]. The upregulation of these genes in highly proliferative cancer cells is believed to be a general adaptive response to chronic replication stress, activating replication origins to compensate for DNA replication fork stalling and inducing DNA repair to cope with chromosomal breakage. However, increase in the expression of regulatory proteins of DNA damage-sensing and repair pathways contribute to improved DNA repair capacity, increased DNA damage tolerance, and the failure of cell death pathways. Consequently, upregulation of DNA repair genes is a mechanism to promote chemoresistance in tumor cells [3].

The RecQ helicases, described as caretakers of the genome, contribute multiple catalytic activities to DNA replication, repair, transcription, and telomere maintenance [4–6]. In humans, functional defects in RecQ proteins predispose to cancer. At the cellular level, loss of their functions leads to increased DNA damage, genomic instability and enhanced sensitivity to a variety of chemotherapeutic agents. The tumor suppressor roles of RecQ proteins are mediated through their genome caretaker roles; and the evidence that RecQ proteins directly regulate tumorigenesis is scarce. The elevated expression of RecQ helicases in rapidly proliferating cells and in many human cancers suggests possible roles in resistance to DNA-damaging agents and their expression levels may predict the responsiveness of cancers to certain chemotherapeutic agents [4]. However, whether or not RecQ helicases are regulated at the mRNA and/or protein level to provide cellular protection against genotoxic stress is poorly known [7].

Here we wanted to investigate whether DNA damage modulates the expression of RECQ1, a RecQ family member critical for DNA repair mechanisms that restore productive replication following stress and prevent genomic instability [8]. *RECQ1* (also known as *RECQL* or *RECQL1*) was recently identified as a breast cancer susceptibility gene [9, 10]. Previous studies have shown that *RECQ1* is upregulated in rapidly dividing cells and its expression is higher in many cancer cell lines as compared to normal cells [11]. Furthermore, *RECQ1* silencing reduces proliferation of cancer cells and suppresses tumor growth in mouse *xenograft* models [12, 13]. RECQ1 can contribute to tumor development and progression by regulating the expression of key genes that promote cancer cell migration, invasion and metastasis [14, 15]. Indeed, *RECQ1* is frequently over-expressed and amplified in many cancer samples (http://www.cbioportal.org/public-portal); and altered *RECQ1* expression is correlated with patient's response to therapy [16–20]. Consistent with this, suppression of *RECQ1* expression in mice and human cells is manifested as constitutively elevated sister chromatid exchange, chromosomal breakage, and increased sensitivity to ionizing radiation [21, 22]. RECQ1 is critical for telomere maintenance [23, 24], restores replication fork progression following stress [25–27], participates in DNA double strand break repair [28], responds to oxidative DNA damage [29, 30], and performs a mechanistic role in base excision repair (BER) pathway which removes chemical alterations to DNA bases such as oxidation and alkylation [31]. Thus, we hypothesized that overexpression of *RECQ1* may provide a survival advantage to cancer cells by promoting the ability of cancer cells to tolerate genotoxic stress.

Herein, we demonstrate that *RECQ1*, the gene encoding the most abundant RecQ family protein in humans, is upregulated upon DNA damage in a p53-dependent manner. These results provide novel insight into regulation of *RECQ1* expression and its role in DNA damage response. As RECQ1 efficiently protects cells from genomic instability through repair of DNA lesions including those induced by alkylating and other chemotherapeutic agents, elevated RECQ1 expression in tumor cells may provide resistance to anticancer drugs.

## RESULTS

### Genotoxic stress upregulates *RECQ1* expression

**Figure 1:**
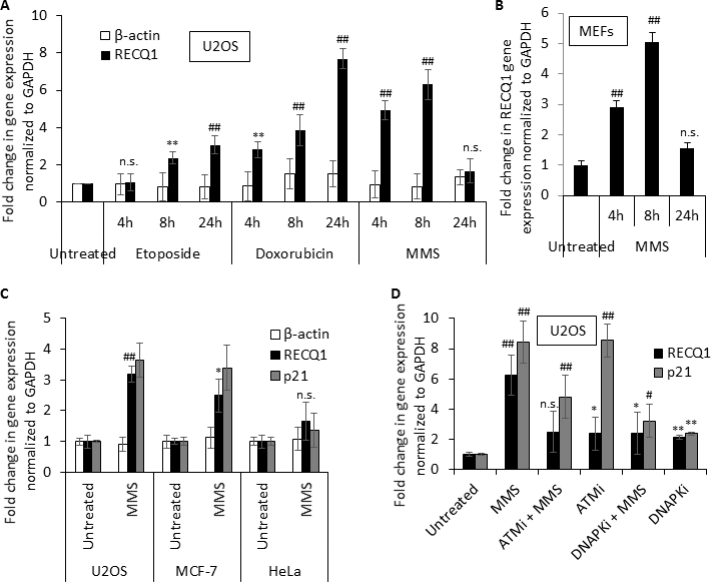
Genotoxic stress upregulates *RECQ1* expression (**A**) Summary of quantitative-PCR data on *RECQ1* mRNA in U2OS cells that were either untreated or treated with etoposide (1 μM), doxorubicin (500 nM) or MMS (1 mM) for 4, 8 or 24 h. Change in β*-actin* mRNA was measured as an additional house-keeping control. (**B**) MMS treatment also upregulates *RECQ1* in mouse embryonic fibroblasts (MEFs). (**C**) MMS induced upregulation of *RECQ1* mRNA is not cell line specific and correlates with upregulation of *p21*, an established p53 target. U2OS, MCF-7, or HeLa cells were untreated or treated with MMS (1 mM) for 4 h. Fold-change in gene expression compared to untreated and normalized to *GAPDH* is shown. (**D**) MMS induced upregulation of *RECQ1* mRNA in U2OS cells is dependent on activities of ATM and DNA-PK. U2OS cells were untreated or treated with pharmacological inhibitors of ATM (ATMi; 10 μM) or DNA-PK (DNA-PKi; 10 μM) for 16 h prior to treatment with MMS (1 mM, 4 h). Fold-change in gene expression compared to untreated and normalized to *GAPDH* is shown. Values are average of three independent experiments and standard deviation is indicated by error bars. Statistical significance of *RECQ1* expression changes in untreated versus treatment groups is indicated as *p < 0.05; ^#^*p* < 0.01; **p < 0.005; ^##^*p* < 0.001; or n. s., non-significant.

To test whether genotoxic stress modulates RECQ1 expression, we first measured *RECQ1* mRNA levels in U2OS (osteosarcoma) cells that were either untreated or treated with etoposide (1 μM), doxorubicin (500 nM) or methylmethanesulfonate (MMS, 1 mM) for 4, 8 or 24 h (Figure 1A). Quantitative RT-PCR (qRT-PCR) analysis demonstrated increased *RECQ1* mRNA levels (2- to 8-fold) in response to these treatments. The kinetics and magnitude of the induction varied for each genotoxic agent. For etoposide and doxorubicin, highest level of *RECQ1* mRNA was observed after 24 h (Figure 1A). As compared to untreated cells, U2OS cells grown for 24 h in the presence of etoposide and doxorubicin displayed about 3- and 8-fold increase in *RECQ1* mRNA, respectively. Treatment with MMS however resulted in an early induction of *RECQ1* mRNA and ~5-fold increase was observed at 4 h following MMS treatment (Figure 1A). In contrast to *RECQ1* mRNA, these treatments did not change β*-actin* mRNA levels. The MMS (1 mM, 4 h) triggered upregulation of *RECQ1* mRNA (3- to 5-fold) was also observed in mouse embryonic fibroblasts (Figure 1B). Treatment with MMS (1 mM, 4 h) also resulted in a significant increase > 2.5-fold (*p* < 0.05) in *RECQ1* mRNA in MCF7 cells (breast cancer) similar to U2OS cells but not in HeLa (cervical carcinoma) cells (Figure 1C).

An important factor involved in transcriptional regulation of genes after DNA damage is p53, which becomes activated by the ATM/ATR pathway upon DNA damage [32]. Analysis of *RECQ1* mRNA levels in U2OS cells treated with MMS indicated a correlation with induction of the canonical p53 target *p21* (*CDKN1A*) suggesting that p53 status may influence DNA damage triggered upregulation of RECQ1 (Figure 1C). Treatment of cells with MMS activates ATM by single strand breaks arising during the repair of alkylating DNA lesions [33]. Similarly, DNA-PK has also been implicated in MMS mediated DNA damage response [34]. To test whether DNA-damage triggered increase in *RECQ1* expression requires activities of ATM and DNA-PK, we measured *RECQ1* mRNA in U2OS cells in the presence of pharmacological inhibitors of ATM (ATMi, Ku55933) or DNA-PK (DNA-PKi, Nu7026) prior to MMS exposure. Incubation of U2OS cells with ATMi or DNA-PKi (10 μM, 16 h) alone resulted in about 2-fold increase in *RECQ1* mRNA (Figure 1D) perhaps due to increased DNA damage load upon inhibiting the activities of these critical signaling kinases. Treatment with MMS failed to induce a significant increase in *RECQ1* mRNA when the ATM or DNA-PK activity was inhibited (Figure 1D) indicating that both ATM and DNA-PK contribute to the observed *RECQ1* upregulation following MMS induced genotoxic stress.

### DNA damage induced upregulation in *RECQ1* expression is p53-dependent

**Figure 2:**
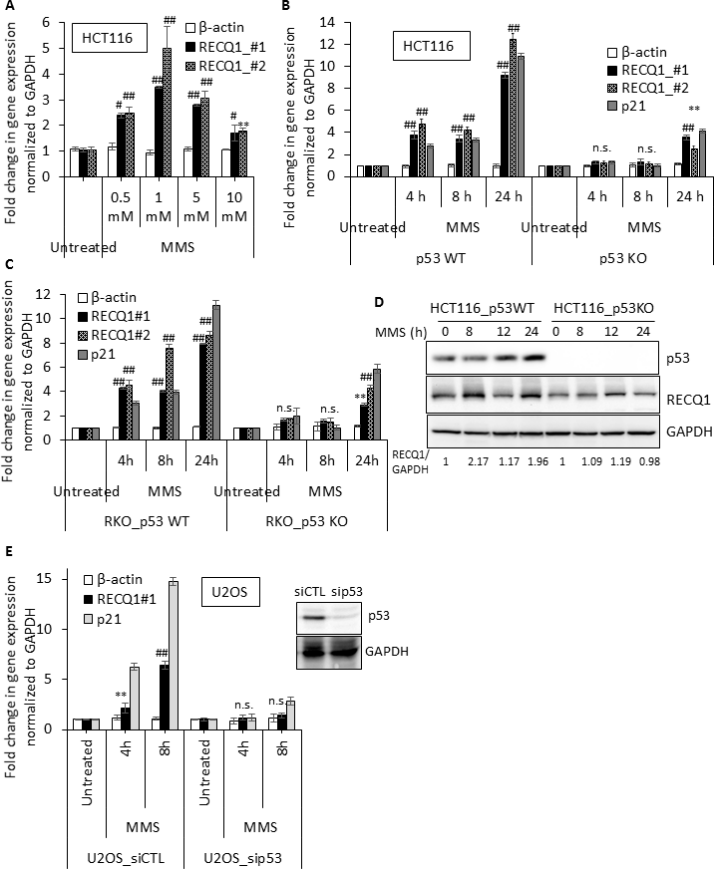
MMS induced upregulation of RECQ1 is p53 dependent (**A**) *RECQ1* expression is upregulated in response to MMS treatment in HCT116 cells expressing wild-type p53. Cells were either untreated or treated for 4 h with the indicated dose of MMS. Fold-change in gene expression compared to untreated and normalized to *GAPDH* is shown. Two primer sets (RECQ1 #1, and RECQ1 #2) were used for measuring *RECQ1* mRNA. β*-actin* served as an additional housekeeping control. (**B**) Isogenic HCT116 cells expressing either the wild-type p53 (p53 WT) or knockout for p53 (p53 KO) were exposed to MMS (1 mM) for indicated time period and the fold-change in mRNA expression of *RECQ1* and *p21* compared to untreated as measured by qPCR is shown. (**C**) Fold-change in mRNA expression of *RECQ1* and *p21* compared to untreated in isogenic p53WT and p53KO RKO cells is shown. (**D**) Following treatment with MMS (1 mM) for indicated time, expression of RECQ1 and p53 proteins was determined by Western blot analysis of whole-cell extracts. GAPDH was used as a loading control and fold-change in RECQ1 protein expression normalized to GAPDH is indicated. (**E**) U2OS cells, 42 h after transfection with control siRNA or p53 siRNA, were exposed to MMS (1 mM) for indicated time period and the fold-change in mRNA expression of *RECQ1* and *p21* compared to untreated cells as measured by qPCR is shown. β*-actin* served as an additional housekeeping control. Knockdown of p53 protein level is shown by Western Blot. For all the qPCR data, values are average of three independent experiments and standard deviation is indicated by error bars. Statistical significance of *RECQ1* expression changes in untreated versus treatment groups is indicated as *p < 0.05; ^#^*p* < 0.01; **p < 0.005; ^##^*p* < 0.001; or n. s., non-significant.

The tumor suppressor p53 functions as a master transcriptional regulator of cellular responses to genotoxic stress by inducing cell cycle arrest or apoptosis [35]. To test whether p53 is involved in *RECQ1* upregulation, we analyzed the MMS-triggered induction of *RECQ1* mRNA in isogenic colon cancer cell lines proficient or deficient for p53. Treatment of p53-proficient HCT116 cells with MMS (4 h) resulted in a dose dependent increase in *RECQ1* mRNA (Figure 2A) as measured by qRT-PCR using two independent primer sets; in contrast, no significant change in β-actin mRNA was observed in response to MMS treatment (Figure 2A). Because we observed maximum induction of *RECQ1* mRNA at 1 mM MMS, we chose this dose of MMS to compare *RECQ1* mRNA levels in p53-proficient (p53 wild-type, p53WT) HCT116 cells and p53-deficient (p53 knockout, p53KO) HCT116 cells. We found that *RECQ1* mRNA was induced only in p53WT-HCT116 cells; p53KO-HCT116 cells did not upregulate *RECQ1* up to after 8 h exposure to MMS (Figure 2B). As a positive control for p53-target, we also observed induction of *p21* mRNA specifically in p53WT-HCT116 cells. In p53WT-HCT116 cells, we observed > 10-fold increase in *RECQ1* mRNA after 24 h of MMS treatment. Notably, p53KO-HCT116 cells displayed a mild (~4-fold) increase in *RECQ1* mRNA levels at 24 h (Figure 2B). This induction is likely due to p53-independent stress response since we also observed *p21* induction, as shown previously [36], in p53KO-HCT116 cells at 24 h treatment (Figure 2B). Notably, this p53-dependent upregulation of *RECQ1* and *p21* mRNA was also observed in p53-proficient but not isogenic p53-deficient RKO cells (Figure 2C). Consistent with the increase in *RECQ1* mRNA, MMS treatment also resulted in a p53-dependent increase in RECQ1 protein in HCT116 cells (Figure 2D). Furthermore, U2OS cells transfected with p53 siRNA failed to upregulate *RECQ1* mRNA and the positive control *p21* mRNA, following MMS (1 mM) treatment (Figure 2E). Collectively, these experiments demonstrate a clear p53-dependence for MMS-induced upregulation of *RECQ1* mRNA.

**Figure 4:**
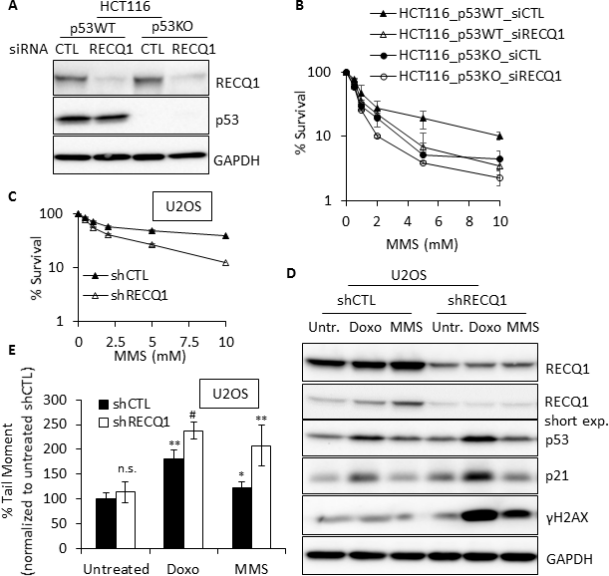
RECQ1 promotes DNA repair and survival after MMS treatment (**A**) Western blot showing siRNA knockdown of RECQ1 in p53-proficient and p53-deficient HCT116 cells. (**B**) RECQ1-depletion and p53 loss have synergistic effect on survival. Following 48 h of siRNA transfection, p53WT and p53KO-HCT116 cells were exposed to MMS for 24 h and subsequently grown for 24 h in drug-free medium. Surviving fraction compared to untreated was determined by cell count. Knockdown of RECQ1 was confirmed by Western blotting as shown. (**C**) U2OS cells stably transduced with a control (shCTL) or RECQ1 (shRECQ1)-specific shRNA were exposed to MMS for 24 h and subsequently grown for 24 h in drug-free medium. Surviving fraction compared to untreated was determined by cell count. (**D**) Western blot analysis of whole cell extracts of stable control and RECQ1 knockdown U2OS cells, untreated or treated with doxorubicin (1 μM) or MMS (1 mM) for 4 h. A short exposure of RECQ1 Western blot is also included. (**E**) DNA double strand breaks in control or RECQ1-depleted cells. Neutral Comet Assay was used to determine tail moment as a measure of double strand breaks in stable control and RECQ1 knockdown U2OS cells, untreated or treated with doxorubicin (1 μM) or MMS (1 mM) for 4 h. Mean tail moment of untreated shCTL was used to normalize the data and is shown as 100%. Statistical significance of difference in tail moment as compared to untreated shCTL is indicated as *p < 0.05; ^#^*p* < 0.01; **p < 0.005 or n. s., non-significant.

The basal levels of *RECQ1* mRNA in p53-deficient HCT116 and RKO cells were 0.6- and 0.8-fold as compared to their p53-proficient isogenic counterparts, indicating that basal *RECQ1* expression level may also be p53-dependent (Supplementary Figure 1). However, basal RECQ1 protein levels were comparable in p53-WT and p53-KO HCT116 cells (Figure 4A). We also note that the MMS-induced upregulation of RECQ1 protein did not follow the same kinetics as the mRNA in p53-proficient HCT116 (Figure 2D). At this point, it is difficult to correlate the regulation at the mRNA level to the changes in protein levels. It is possible that RECQ1 is regulated both at the transcriptional and posttranscriptional levels. We also analyzed mRNA levels of all five human RecQ homologs in HCT116 and RKO cells for DNA damage and p53 dependence (Supplementary Figure 2). Consistent with a previous report of transcriptional repression of RECQ4 by p53 [37], MMS-treated p53-deficient cells displayed elevated *RECQ4* mRNA (Supplementary Figure 2). We did not observe significant changes in expression of *BLM* or *RECQ5* in response to MMS treatment, however *WRN* expression was upregulated in p53-dependent fashion (Supplementary Figure 2). In p53WT-HCT116 cells, about 6-fold and 2-fold higher *WRN* mRNA was measured at 4 and 24 h MMS treatment (1 mM) as compared to untreated cells. In contrast, about 4-fold and 10-fold increase in *RECQ1* mRNA was measured at 4 and 24 h MMS treatment (1 mM), respectively (Supplementary Figure 2). Distinct kinetics and p53-dependence of *RECQ1*, *WRN* and *RECQ4* mRNA upregulation suggests that encoded proteins participate at different steps and/or pathways for cellular response to MMS treatment.

### The RECQ1 promoter-luciferase reporter is responsive to p53

**Figure 3:**
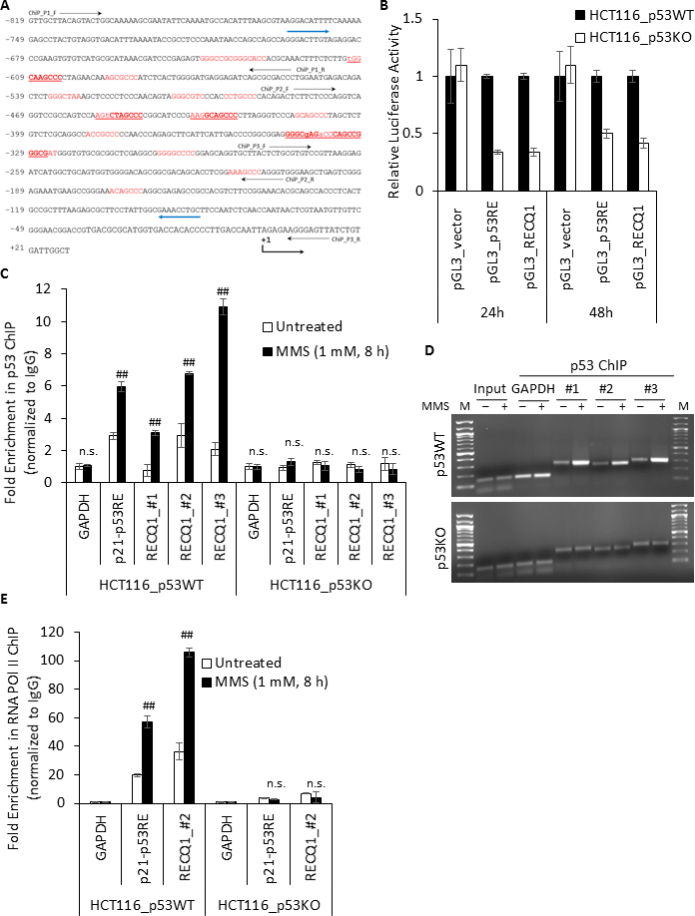
p53 is enriched at the *RECQ1* promoter following MMS treatment (**A**) Partial sequence of the *RECQ1* promoter (−819 bp to +21 bp) is shown with potential p53-binding sites predicted by Promo 3.0.2 indicated in red, those predicted by Tp53 database are underlined, and those predicted by both Promo 3.0.2 and Tp53 are indicated in bold. Transcriptional start site is indicated as +1. The position of primer used for PCR-cloning of a 625 bp fragment from *RECQ1* promoter in pGL3-Basic for luciferase assay is indicated by blue arrow. Three primer sets used for ChIP-qPCR analyses of p53 binding are indicated. (**B**) Dual Luciferase Assay shows p53-dependent transcriptional activation of a 625bp fragment from *RECQ1* promoter region; pGL3_p53RE served as a positive control and pGL3_vector as a negative control. The relative luciferase activity was first determined by ratio of firefly and renilla luciferase activity for each sample in p53-WT and p53-KO HCT116 cells, and the relative promoter activity in p53-KO was calculated using the relative luciferase activity from p53-WT cells transfected by each pGL3-basic construct as a reference of 1. Bars indicate mean values plus standard deviation of three independent experiments. (**C**) MMS-induced enrichment of p53 to *RECQ1* promoter. HCT116 cells, p53-WT or p53-KO, untreated or treated with MMS (1 mM, 8 h), were processed for ChIP using a p53-specific antibody. ChIP experiments with rabbit IgG served as negative control. ChIP-qPCR of immunoprecipitated DNA with three probes specific for *RECQ1* promoter sequence containing predicted p53 binding sites (#1, #2, and #3, and as shown in A). Binding of p53 to *p21* promoter containing p53RE served as a positive control. Fold enrichment over IgG was determined and is shown for each primer pair for the ChIP. Relative occupancy at *RECQ1* and *p21* promoter sequence versus a negative control site DNA containing *GAPDH* shows MMS treatment induced enrichment of p53. Statistical significance of enrichment in untreated versus treatment groups is indicated. Results are expressed as means ± SEM for at least three independent experiments. (**D**) A representative agarose gel of the amplified DNA immunoprecipitated with p53 antibody shows MMS-induced enrichment of p53 to *RECQ1* promoter whereas MMS treatment did not change *GAPDH* abundance in p53 ChIP. M, DNA size marker. (**E**) Enhanced recruitment of RNA POL II to *RECQ1* promoter following MMS treatment. HCT116 cells, p53-WT or p53-KO, untreated or treated with MMS (1 mM, 8 h), were processed for ChIP using a RNA POL II-specific antibody or rabbit IgG. Binding of RNA POL II to *RECQ1* promoter sequence and *p21* promoter was measured by qPCR of immunoprecipitated DNA. Fold enrichment over IgG was determined and is shown for each primer pair for the ChIP. Relative occupancy at *RECQ1* and *p21* promoter sequence versus a negative control non-promoter *GAPDH* sequence shows MMS treatment induced enrichment of RNA POL II. Statistical significance of enrichment in untreated versus treatment groups is indicated. Results are expressed as means ± SEM for at least three independent experiments.

Having identified that MMS-induced *RECQ1* upregulation is p53-dependent, we sought to determine if *RECQ1* is a direct target of p53. Transcription regulation by p53 is enacted through its binding to the consensus sequence motif RRRCWWGYYY(N_0–13_)RRRCWWGYYY where R is a purine, W is an adenine or thymine, Y is a pyrimidine and N is any base [38]. Additionally, p53 can bind to non-canonical response elements (REs) or half and three-quarter consensus sequences to mediate transactivation of several genes including those involved in DNA repair responses [39]. Analysis using the IARC Tp53 database (http://p53.iarc.fr/TargetGenes.aspx?&sq=p53+response+element) suggested p53-binding sites in the *RECQ1* promoter. We searched for the potential binding sites of p53 in a region ~1 kb upstream and downstream of the human *RECQ1* transcriptional start site using Promo 3.0.2 online tool (http://alggen.lsi.upc.es/cgi-bin/promo_v3/promo/promoinit.cgi?dirDB=TF_8.3) [40] and identified multiple putative p53-binding sites, majority of which are located within 819 bp upstream of transcription start site and 21 bp of exon 1 (Figure 3A). Of note, and as previously reported for p53, these predicted sites deviate from the consensus p53RE. To test the hypothesis that p53 promotes *RECQ1* transcription, we first utilized a luciferase-reporter system employing the pGL3-Basic promoterless vector or pGL3-carrying a 625 bp *RECQ1* promoter fragment containing the putative p53 binding sites and analyzed luciferase expression in the p53-proficient and p53-deficient HCT116 cells. Our reporter assay also included a pGL3-p53RE that contained 11 consensus p53 response elements as positive control [41]. We observed that at both 24 and 48 h after transfection, luciferase expression from the *RECQ1* promoter-luciferase reporter was significantly higher in p53WT- HCT116 cells as compared to p53KO- HCT116 cells (Figure 3B). As a positive control, luciferase expression of pGL3-p53RE was also significantly reduced in p53KO-HCT116 cells whereas the luciferase activity of pGL3-empty vector remained comparable in p53-deficient or p53-proficient cells (Figure 3B). This data indicates that basal *RECQ1* promoter activity is driven by p53 and is consistent with our data showing that endogenous basal *RECQ1* mRNA levels are higher in p53WT-HCT116 cells as compared to p53KO-HCT116 cells (Supplementary Figure 1).

### DNA damage recruits p53 to the RECQ1 promoter

We next employed chromatin immunoprecipitation (ChIP) experiments in conjunction with qPCR to determine if endogenous p53 associates with the predicted binding sites in the *RECQ1* promoter. Cross-linked chromatin from p53-proficient or p53-deficient HCT116 cells, untreated or treated with MMS (1 mM, 8 h) was immunoprecipitated with a control IgG or specific antibody against p53 (DO-1). Following cross-link reversal, the immunoprecipitated chromatin was subjected to qPCR to determine the enrichment of RECQ1-promoter sequence using three independent primer sets spanning the predicted p53 binding sites within *RECQ1* promoter. In untreated p53WT- HCT116 cells, we found ~2-fold enrichment of p53 at the *RECQ1* promoter containing putative p53 binding sequences (Figure 3C). Treatment with MMS resulted in a marked increase in p53 binding at the *RECQ1* promoter; we observed ~3-, 7-, and 11-fold enrichment in p53 binding at RECQ1 promoter site 1, 2 and 3, respectively in p53WT-HCT116 cells (Figure 3C). As a positive control for the p53-ChIP assay, we confirmed p53 binding to a known p53RE of *p21* [41], a downstream target of p53, in p53WT-HCT116 cells (Figure 3C). MMS treatment also resulted in 6-fold enrichment of p53 at the *p21* promoter as compared to 2-fold enrichment in untreated p53WT- HCT116 cells (Figure 3C). In contrast, the *RECQ1* promoter or the *p21* promoter was not enriched in the p53-ChIP material in p53KO-HCT116 cells indicating that the observed enrichment of the *RECQ1* and *p21* promoter in the p53-ChIP material in HCT116-p53WT cells was specific to p53 (Figure 3C, 3D). In untreated or MMS-treated condition, we did not observe enrichment of a negative control genomic region corresponding to *GAPDH* in the p53-ChIP over IgG-ChIP, (Figure 3C, 3D) demonstrating specificity of the interaction between p53 and the p53-response elements in the RECQ1 promoter. Chromatin immunoprecipitation with RNA polymerase (Pol) II antibody demonstrated a p53-dependent and significantly enhanced recruitment of RNA Pol II to the promoter regions of *RECQ1* and *p21* upon MMS treatment suggesting transcriptional upregulation (Figure 3E). Binding of p53 to the *RECQ1* promoter and the enhanced binding following MMS treatment was also observed in U2OS cells (Supplementary Figure 3A). We also observed binding in these regions of the *RECQ1* promoter corresponding to the p53 ChIP-seq peak in data from U2OS cells published by others [42] (Supplementary Figure 3B–3D). Our results suggest *in vivo* association of p53 with *RECQ1* promoter is enriched upon MMS-induced DNA damage. Overall, the presence of p53 binding site, and the observed MMS-induced enrichment of p53 at the *RECQ1* promoter is consistent with the hypothesis that p53 mediates the upregulation of *RECQ1* after DNA damage.

### RECQ1 promotes DNA repair and survival following MMS-treatment

Given the well-established roles of p53 in genotoxic stress response, we sought to determine the effect of RECQ1-depletion in p53-proficient and p53-deficient HCT116 cells on survival after MMS treatment. We transfected these cells with a control (CTL) siRNA or RECQ1 siRNAs (20 nM) for 48 h and validated significant knockdown of RECQ1 protein (> 90%) by immunoblotting (Figure 4A). Following 48 h of transfection, CTL or RECQ1 siRNA-transfected cells were exposed to increasing concentrations of MMS for 24 h and cell survival was measured by trypan blue exclusion assay following a 24 h recovery in drug free medium (Figure 4B). As reported previously [43], p53KO-HCT116 cells were more sensitive to MMS treatment than p53WT-HCT116 cells. As compared to CTL siRNA transfected cells, RECQ1-depletion in p53WT-HCT116 cells also resulted in increased MMS sensitivity; however, RECQ1-depletion in p53KO-HCT116 cells caused maximum sensitivity to MMS (Figure 4B). This synergistic effect on survival upon concurrent deficiency of RECQ1 and p53 indicates their parallel roles in DNA damage response to MMS. It is also likely that compensatory mechanisms operate in the combined absence of RECQ1 and p53 since the depletion of RECQ1 in p53-KO cells did not show a dramatic reduction in survival.

Similar to our observation in HCT116 cells, U2OS cells stably transduced with a RECQ1 shRNA were more sensitive than those transduced with a CTL (luciferase)-shRNA over a range of MMS concentration tested (Figure 4C). This data demonstrates that the increased sensitivity to MMS is not cell-type specific and is sustained in cells depleted of RECQ1 over a longer period of time. MMS is known to produce heat-labile DNA damage repaired by base excision repair, but no detectable *in vivo* DNA double strand breaks [44]. DNA alkylation due to MMS also blocks replication fork elongation in mammalian cells, causing formation of replication-associated DNA lesions [45]. The cellular level of RECQ1 protein modulates overall DNA damage [22] and RECQ1 functions in replication restart following stress [25]. Thus, we next examined γH2AX as a surrogate marker of DNA double strand breaks in CTL and RECQ1-knockdown U2OS cells exposed to MMS (1 mM, 4h) or Doxorubicin (1 μM, 4 h), a topoisomerase II-stabilizing drug known to cause double strand breaks in DNA [46]. At the protein level, increased RECQ1 levels were observed in control U2OS cells upon treatment with MMS and doxorubicin (Figure 4D); and this increase was more readily noticeable in short exposure due to constitutively high abundance of RECQ1 protein in total cell lysate used in Western Blots. The increased RECQ1 protein is consistent with the observed upregulation of *RECQ1* mRNA in U2OS cells (Figure 1A). In control U2OS cells, MMS treatment did not result in significant double strand breaks as detected by γH2AX signal. In contrast, RECQ1-knockdown U2OS cells demonstrated greater γH2AX signal after treatment with MMS (Figure 4D). As compared to control U2OS cells, RECQ1-knockdown cells also exhibited significantly greater γH2AX after doxorubicin treatment (Figure 4D). As reported previously [46], doxorubicin treatment stabilized p53 and increased p21 levels indicating transcriptional activation; RECQ1-knockdown cells displayed greater signal for p53 and p21 proteins as compared to control U2OS cells (Figure 4D). Since γH2AX is formed in response to direct or indirectly-produced double strand breaks, the presence of double strand breaks was also measured using a neutral comet assay. We determined tail moment, a measure of the amount and distance of DNA migration, in control and RECQ1-knockdown U2OS cells. As compared to untreated condition, treatment with Doxorubicin (1 μM, 4 h) or MMS (1 mM, 4 h) resulted in increased tail moment in U2OS cells (Figure 4E). Consistent with the observed increase in γH2AX, RECQ1-knockdown U2OS cells exhibited increased mean tail moment as compared to control U2OS cells, indicating higher levels of double strand breaks when exposed to doxorubicin or MMS in neutral comet assays (Figure 4E).

Overall, increased accumulation and transactivation of p53, and increased double strand breaks are consistent with the observed sensitivity of RECQ1-deficienct cells to MMS as shown here and doxorubicin as recently reported [47].

### *RECQ1* expression positively correlates with cellular resistance to clinically relevant DNA damaging agents

**Figure 5:**
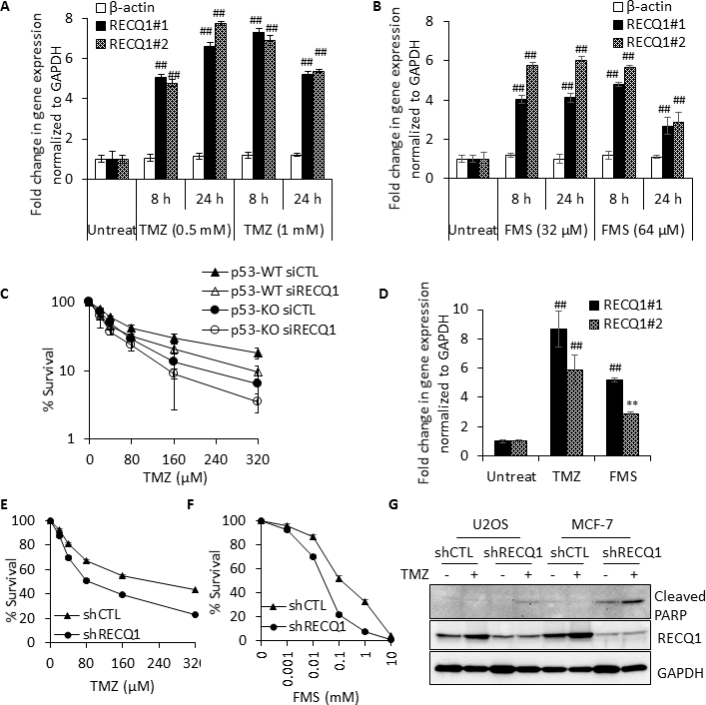
*RECQ1* expression is upregulated by the chemotherapeutic drugs that alkylate DNA and positively correlates with cellular resistance to these treatments (**A**, **B**) *RECQ1* expression is upregulated in response to Temozolomide (TMZ) or Fotemustine (FMS) treatment of HCT116 cells expressing wild-type p53. *RECQ1* mRNA expression was measured using two independent primer sets; fold-change in expression compared to untreated and normalized to *GAPDH* is shown. β*-actin* served as an additional housekeeping control. Values are average of three independent experiments and standard deviation is indicated by error bars. Statistical significance of *RECQ1* expression changes in untreated versus treatment groups is indicated as *p < 0.05; ^#^*p* < 0.01; **p < 0.005; ^##^*p* < 0.001; or n. s., non-significant. (**C**) Following 48 h of control or RECQ1 siRNA transfection, p53WT and p53KO-HCT116 cells were exposed to increasing dose of TMZ for 24 h and subsequently grown for 24 h in drug-free medium. Surviving fraction compared to untreated was determined by cell count. Knockdown of RECQ1 was confirmed by Western blotting (not shown). Surviving fraction values are the mean ± SEM from three independent experiments. (**D**) *RECQ1* expression is also upregulated in U2OS cells following treatment with TMZ (1 mM, 6 h) or FMS (32 μM, 6 h). Fold-change in expression compared to untreated and normalized to *GAPDH* is shown. Values are average of three independent experiments and standard deviation is indicated by error bars. Statistical significance of change in *RECQ1* expression compared to untreated is indicated. (**E**, **F**) Stable control (shCTL) and RECQ1 knockdown (shRECQ1) U2OS cells were exposed to increasing dose of TMZ or FMS for 24 h and subsequently grown for a further 24 h in drug-free medium. The graphs show the cellular surviving fractions measured at different doses of drug treatment in control and RECQ1-depleted cells. Surviving fraction values are the mean ± SEM from three independent experiments. (**G**) Whole cell extracts prepared from stable control and RECQ1 knockdown U2OS and MCF7 cells cultured for 5 days in the absence or presence of TMZ (100 μM) were subjected to Western blot analysis of cleaved PARP for apoptosis. Knockdown of RECQ1 was verified by Western blotting. TMZ treatment lead to increase in RECQ1 protein level in both U2OS and MCF7 cells transduced with control shRNA.

RECQ1 deficiency leads to genomic instability and sensitivity to a range of genotoxins [4, 5]. Given the sensitivity of RECQ1-deficient cells to MMS, we first examined whether *RECQ1* expression is upregulated at the mRNA level after treatment with clinically relevant alkylating agents Temozolomide (TMZ) and Fotemustine (FMS), both of which are used as cancer chemotherapeutics [48]. Following 8 h treatment of p53WT-HCT116 cells with TMZ, > 4-fold and 6-fold increase in *RECQ1* mRNA was observed in cells treated with 0.5 mM and 1 mM TMZ, respectively (Figure 5A). Treatment with FMS also upregulated *RECQ1* mRNA in HCT116 cells, and a comparable increase (> 4-fold) in *RECQ1* mRNA was observed after 8 h treatment with 32 μM or 64 μM FMS (Figure 5B). As observed for the MMS sensitivity, p53KO-HCT116 cells were more sensitive for TMZ and RECQ1-depletion led to increased sensitivity in both p53-proficient and p53-deficient HCT116 cells (Figure 5C). Moreover, treatment with TMZ (1 mM, 8 h) or FMS (32 μM, 8 h) also upregulated *RECQ1* mRNA in U2OS cells (Figure 5D); and RECQ1-knockdown cells were more sensitive than control U2OS cells over a range of TMZ and FMS concentration tested (Figure 5E, 5F). Presence of TMZ (100 μM, 5 days) in growth medium resulted in increased RECQ1 protein level in control U2OS and MCF7 cells, and led to cleaved PARP in RECQ1 knockdown U2OS and MCF7 cells indicating enhanced apoptosis (Figure 5G). These results collectively demonstrate that DNA alkylating agents including the laboratory agent MMS and chemotherapeutics TMZ or FMS induce upregulation of *RECQ1* mRNA, and RECQ1 expression level correlates with cellular sensitivity to these treatments. As compared to control untreated U2OS cells, *RECQ1* mRNA was also upregulated following treatment with chemotherapeutics 5-fluoro uracil (5-FU), gemcitabine, camptothecin as well as the laboratory carcinogen methylnitronitrosoguanidine (MNNG) and environmental mutagen benzo[a]pyrene (Supplementary Figure 4). This is consistent with previous studies where RECQ1-depletion increased cellular sensitivity to camptothecin and benzo[a]pyrene [22, 49].

### Genotoxic stress-induced upregulation of *RECQ1* mRNA expression in MDA-MB-231 cells

**Figure 6:**
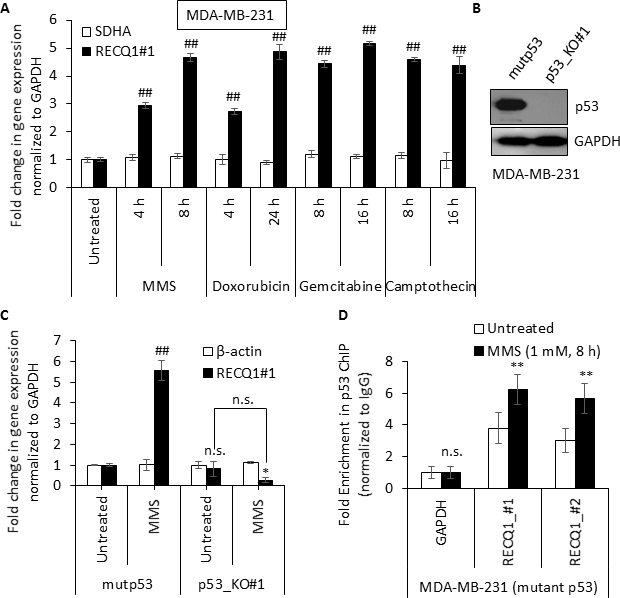
DNA damage induced upregulation of *RECQ1* expression in MDA-MB-231 cells expressing mutant p53 (**A**) RNA was isolated from MDA-MB-231 cells that were untreated or treated with MMS (1 mM), doxorubicin (1 μM), gemcitabine (2 μM) or camptothecin (1 μM) as indicated. Fold-change in *RECQ1* mRNA expression compared to untreated and normalized to *GAPDH* is shown. *SDHA* served as an additional housekeeping control. Values are average of three independent experiments and standard deviation is indicated by error bars. Statistical significance of change in *RECQ1* expression compared to untreated is indicated. (**B**) Western Blots showing loss of p53 protein in TP53 knockout (p53_KO#1) MDA-MB-231 clone and another clone with unchanged level of p53 (mutp53). GAPDH was used as a loading control. (**C**) Isogenic MDA-MB-231 cells expressing with the mutant p53 (mutp53) or knockout for p53 (p53_KO#1) were exposed to MMS (1 mM) for 8 h and the fold-change in mRNA expression of *RECQ1* and β*-actin* compared to untreated and normalized to *GAPDH* is shown. Statistical significance of change in *RECQ1* expression compared to untreated mup53 is indicated. (**D**) MMS-induced enrichment of mutant p53 to *RECQ1* promoter in MDA-MB-231 cells. MDA-MB-231 cells, untreated or treated with MMS (1 mM, 8 h), were processed for ChIP using a p53-specific antibody. ChIP experiments with rabbit IgG served as negative control. ChIP-qPCR of immunoprecipitated DNA with primers specific for *RECQ1* promoter sequence containing predicted p53 binding sites (#1 and #2, as shown in 3A) was performed. Fold enrichment over IgG was determined and is shown for each primer pair for the ChIP. Statistical significance of enrichment in untreated versus treatment groups is presented. Results are expressed as means ± SEM for at least three independent experiments.

p53 is a well-known tumor suppressor and p53 mutations are by far the most common in human cancer, including breast cancer. MDA-MB-231 human breast cancer cells express mutant p53 (p53R280K) that confers a “gain of function” including chemotherapy resistance, metabolic deregulation, and increased metastasis. Unlike wild-type p53 that primarily mediates its effects through sequence-specific DNA binding to cognate p53 response elements located in the promoters of p53 target genes, the mutant p53 protein generally has diminished wild-type p53 activity but it regulates gene expression via interactions with other transcription factors [50–53]. Interestingly, we found that DNA damage also upregulated *RECQ1* mRNA in MDA-MB-231 cells (Figure 6A). To determine whether mutant p53 affected the expression of *RECQ1*, we utilized the CRISPR/Cas9 technology to knockout mutant p53 in MDA-MB-231 cells (Supplementary Figure 5). After confirming successful targeted deletion by Sanger sequencing (data not shown), we performed immunoblotting. As shown in Figure 6B, mutant p53 protein was lost in the knockout clone (p53_KO#1) but it was robustly expressed in an isogenic clone in which the mutant p53 gene was intact (mutp53). MMS treatment resulted in a 5-fold increase in *RECQ1* mRNA in the mutp53-expressing clone whereas no significant change in *RECQ1* mRNA was observed in p53_KO#1 clone (Figure 6C). Furthermore, p53-ChIP in MDA-MB-231 cells recovered *RECQ1* promoter regions indicating that mutant p53 associates with the *RECQ1* promoter (Figure 6D). These data indicate that *RECQ1* mRNA is induced by DNA damage in a mutant p53-dependent manner in MDA-MB-231 cells.

### Clinical correlation of RECQ1 expression in human cancer

**Figure 7:**
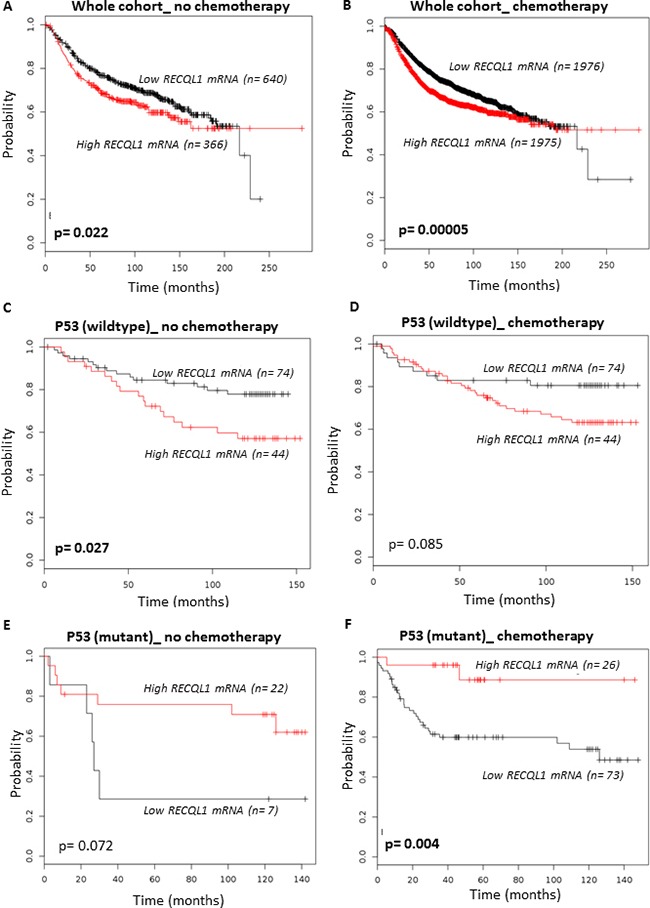
Clinical correlation of *RECQ1* expression with p53 status for survival outcomes in breast cancer Kaplan-Meier curves correlating *RECQ1* mRNA expression and relapse free survival in breast cancer patients from kmplotter.com are shown. (**A**, **B**) Survival of patients, in the whole cohort of 3951 breast tumors, who received no chemotherapy (A) or did receive chemotherapy (B). (**C**, **D**) Survival curves for patients with wild-type p53 who received no chemotherapy (C) or received chemotherapy (D). (**E**, **F**) Survival curves for patients with mutant p53 who received no chemotherapy (E) or received chemotherapy (F).

Germline mutations in *RECQ1* significantly enhance lifetime risk of developing breast cancer [9, 10]. In the Cancer Genome Atlas (TCGA), out of 817 patients analyzed for the Breast Invasive Carcinoma [54], 68 samples showed *RECQ1* mRNA upregulation whereas only 7 showed downregulated *RECQ1* mRNA. In addition, the *RECQ1* gene is affected also by copy number alterations, with 3 patients showing a deep deletion and 17 patients showing an amplification of which 14 also showed *RECQ1* overexpression (Supplementary Figure 6A). In this dataset, carriers of altered *RECQ1* copy number and mRNA expression show a significantly shorter overall survival (Supplementary Figure 6B). Analysis of TCGA data shows significant association of *RECQ1* alterations with p53 mutation (*p* < 0.001). Similarly, *RECQ1* alterations exhibit significant tendency to co-occur with p53 mutations in METABRIC cohort of breast cancer patients where *RECQ1* expression is correlated with patient survival [47]. To investigate whether *RECQ1* mRNA expression predicts response to chemotherapy, we explored a large gene expression data set of 3951 human breast tumors (http://kmplot.com/analysis/index.php?p=service&default=true) [55]. In the whole cohort that received chemotherapy, high *RECQ1* mRNA was associated with poor survival (Figure 7B) (*p* = 0.00005). In patients who received no chemotherapy, high *RECQ1* mRNA was also associated with poor survival (Figure 7A), but this association was less significant (*p* = 0.022). In p53 wild-type tumors, high *RECQ1* mRNA was associated with poor survival in patients who received no chemotherapy (Figure 7C) (*p* = 0.027) and was borderline non-significant in patients who received chemotherapy (Figure 7D) (*p* = 0.085). These data may be consistent with our result from cell lines where we found that in p53 wild-type cells, knockdown of *RECQ1* results in increased sensitivity to DNA damage. Interestingly in p53 mutant tumors, we observed the opposite. Low *RECQ1* mRNA was significantly associated with poor survival in patients who received chemotherapy (Figure 7F) (*p* = 0.004) and was borderline non-significant in patients who received no chemotherapy (Figure 7E) (*p* = 0.072). Taken together, these clinical data suggest that the prognostic and predictive significance of *RECQ1* expression may be influenced by p53 status in breast cancers.

## DISCUSSION

Our results demonstrate upregulation of endogenous RECQ1 upon exposure to DNA damaging agents. We found that the DNA damage induced upregulation of *RECQ1* mRNA is p53-dependent which binds and activates the *RECQ1* promoter. We demonstrate that induction of RECQ1 by DNA damage occurs in p53-proficient HCT116 cells at the level of mRNA and protein. *RECQ1* expression is also upregulated in cells exposed to TMZ, FMS, and doxorubicin; and correlates positively with cell survival following treatment with these agents. Induction of *RECQ1* mRNA by genotoxins was seen in osteosarcoma, breast cancer, and colon cancer cell lines. Our observation that mouse embryonic fibroblasts also upregulated *RECQ1* expression in response to DNA damage is consistent with its DNA repair function and suggests that RECQ1 may protect normal cells from the DNA damaging effects of anticancer drugs. Upregulation of *RECQ1* expression in cancer cells, however, may contribute to chemoresistance. Our findings are consistent with a previous proteome-wide study where etoposide treatment increased the abundance of RECQ1 peptides specifically in p53-proficient cells but not in p53-deficient cells [56]. This is the first report of *RECQ1* as being p53-regulated transcriptional target, although a distinct role for RECQ1 has been described in the maintenance of the genomic stability [57]. RECQ1 catalyzes DNA unwinding and strand annealing [58], and these activities are likely to be important for its role in DNA repair [8]. Together our findings significantly expand on prior observation and indicate that transcriptional regulation of *RECQ1* is potentially involved in an adaptive and/or a protective response to genotoxic stress.

Standard chemotherapy for cancer aims to produce replication stress induced DNA damage thereby promoting death preferentially in rapidly proliferating cancer cells. However, the ability of cancer cells to recognize this damage and initiate DNA repair is an important mechanism for drug resistance and poor therapeutic efficacy [1]. Genetic variations such as mutations, copy number changes, change in mRNA expression, or single nucleotide polymorphisms in DNA repair genes may encode alterations that affect DNA repair function and, therefore, influence individual's risk of cancer development and clinical response to cytotoxic therapies [1, 2]. A polymorphism in *RECQ1*, A159C located in the 3′UTR, is associated with faster tumor progression and significantly reduced survival of pancreatic adenocarcinoma patients who received gemcitabine and radiotherapy [16]. Gemcitabine, a nucleoside analog, disrupts DNA replication and interferes with the homologous recombination repair of DNA, whereas RECQ1 helicase functions are important to restore productive DNA replication [8]. It is possible that the A159C variant allele confers higher *RECQ1* expression, leading to a better repair of the gemcitabine-induced DNA damage and, hence, a poor clinical response. In oral squamous cell carcinoma, *RECQ1* expression correlated with cisplatin resistance [59] and RECQ1-depletion significantly augmented the *in vivo* anticancer effects of the drug cis-platinum (II) diammine dichloride that induce inter-strand cross links in DNA to impair progression of replication forks [19]. In sporadic ER- negative breast tumors, high *RECQ1* expression is associated with poor survival in patients that received anthracycline based chemotherapy; and RECQ1-depletion in breast cancer cells increased doxorubicin chemosensitivity owing to DNA double strand breaks accumulation, S-phase cell cycle arrest and apoptosis [47]. Viziteu et al. recently reported that RecQ helicases are deregulated in hematological malignancies compared to their normal counterparts and expression of *RECQ1* was associated with significantly poor survival [60]. In a subsequent study, *RECQ1* overexpression in multiple myeloma cells conferred resistance against the DNA alkylating drug melphalan and proteasome inhibitor bortezomib, and high expression of *RECQ1* associated with poor prognosis in multiple myeloma patients treated with high dose melphalan [61]. Noteworthy, *RECQ1* was significantly overexpressed in patients with deletion of chromosome 17p which harbors TP53, suggesting that additional factors may regulate *RECQ1* expression [61]. Observed sensitivity of RECQ1-depleted cells to DNA alkylating agents is consistent with a newly identified role of RECQ1 in BER [31] and a previous study where glioblastoma cells depleted of RECQ1 were found to be more sensitive to TMZ treatment [62]. Indeed, deficiency of a BER factor or imbalance in BER enzymatic steps contribute to sensitizing cells to killing effects of alkylating agents [63]. A mechanism to tolerate alkylating adducts is through the activities of translesion DNA polymerases [3]. However, unrepaired DNA adducts in such cases interfere with replication fork progression giving rise to secondary DNA damage including strand breaks [44]. RECQ1 through the repair of stalled replication forks at sites of alkylated bases and its role in BER may serve to prevent chromosome breakage upon exogenous replication stress and DNA damage induced by alkylating agents.

It is yet unclear how the missense mutations that disrupt RECQ1 enzymatic activities and altered (mostly increased) *RECQ1* expression will impact breast cancer prognosis. A survey of Oncomine reveals that *RECQ1* is overexpressed and amplified in many clinical cancer samples versus normal samples [27, 64]. In human cells, RECQ1 is ranked amongst the top 5–25% most abundant proteins in Protein Abundance Database, PaxDb [65]. It can be reasoned that even a 2-fold increase in the basal level of RECQ1 protein in cells that already express it at significantly high level will likely enhance cellular capacity of DNA repair following DNA damage. Given the roles of RECQ1 in resolving replication stress, our results suggest that *RECQ1* overexpression could be a marker of chemoresistance and DNA damage induced upregulation of RECQ1 would be especially important for survival of highly proliferative cancer cells. Targeted inhibition of *RECQ1* expression or pharmacological inhibition of RECQ1 enzymatic activity could potentially enhance therapeutic action of anticancer drugs in p53-deficient tumor cells that are much more reliant than normal cells on pathways that resolve replication stress. Although we found that *RECQ1* mRNA is induced upon DNA damage in the mutant p53-expressing MDA-MB-231 cells and this induction is lost upon targeted deletion of mutant p53, future studies are needed to determine if this is also observed in a panel of cell lines that express gain-of-function mutant p53 proteins. Given the established role of RECQ1 in DNA repair, it will be interesting to examine if the enhanced chemoresistance of mutant p53-expressing cells is mediated, in part, via induction of *RECQ1*.

Our demonstration that RECQ1 is a p53-responsive gene in the context of genotoxic stress suggests that a RECQ1 inhibitor may be beneficial to cancer patients who retain wild-type p53. Notably, and despite significant sequence conservation among the RecQ family members, specific inhibitors of WRN and BLM helicases have been developed [66, 67]. *RECQ1* knockout animals are phenotypically normal [21], indicating that the RECQ1 helicase is not a general regulator of cellular proliferation and aberrant expression in cancer may be acquired on cellular transformation, suggesting targeting RECQ1 could be potentially tumor specific. The p53 tumor suppressor protein is critical in orchestrating the genomic response to stress by transcriptional regulation of genes involved in key cellular processes such as DNA repair, cell cycle arrest, senescence and apoptosis. It would be interesting to test, in future, whether concurrent p53 loss will expose the deleterious effects of genetic knockout of *RECQ1* in mice which might otherwise be compensated in a p53-proficient background. Given the recent associations of *RECQ1* mutations and expression with cancer susceptibility and response to therapy, it will be important to understand the roles of RECQ1 in the context of other relevant and known susceptibility genes in tumor biology.

In summary, our findings illustrate previously unknown regulation of *RECQ1* expression in response to DNA damage and may be useful in understanding the clinical significance of *RECQ1* expression in tumor development and therapeutic response. Identification of synergistic genes and pathways, and characterization of regulatory mechanisms for *RECQ1* expression through the functional studies may instruct alternative therapeutic strategies.

## MATERIALS AND METHODS

### Cell culture and DNA damage treatments

Isogenic pair of p53-wild type (p53WT) and p53-knockout (p53KO) human colon carcinoma cell lines HCT116 and RKO were provided by Dr. B. Vogelstein (The Johns Hopkins Kimmel Cancer Center, Baltimore, MD); human cervical adenocarcinoma HeLa, osteosarcoma U2OS and breast adenocarcinoma MCF7 and MDA-MB-231 cell lines used in this study were purchased from American Type cell culture (ATCC); and mouse embryonic fibroblasts (MEFs) have been described [21]. Cells were maintained in Dulbecco's Modified Eagle's Medium (DMEM) containing 10% fetal bovine serum (FBS). All cells were cultured in a humidified atmosphere containing 5% CO2 at 37°C and routinely checked for mycoplasma contamination using a PCR based assay (Sigma, catalog no. MP0035). MMS, TMZ, FMS, doxorubicin, etoposide, camptothecin, gemcitabine, MNNG, 5-FU, and benzo[a]pyrene, were purchased from Sigma; inhibitors for ATM (Ku55933) and DNA-PK (Nu7026) were purchased from Selleck Chemicals. All stock solutions were made as recommended by the vendors and used for treatment of cells in culture.

### RNAi mediated knockdown of RECQ1 and p53

On-Target plus SMARTpool small interfering RNAs (siRNAs) against RECQ1 or p53 and control siRNAs were purchased from Dharmacon. All siRNA transfections were performed by reverse transfection at a final concentration of 20 nM using Lipofectamine RNAiMAX (Invitrogen) as instructed by the manufacturer. Stable shRNA-mediated knockdown of RECQ1 in U2OS cells was achieved using a lentiviral system [25]. Briefly, lentivirus particles were produced by cotransfecting 293T cells with the pLKO.1 lentiviral shRNA expression vector containing the RECQ1 targeting sequence (5′-GAGCTTATGTTACCAGTTA-3′) or the gene encoding Luciferase (5′-ACGCTGAGTACTTCGAAATGT-3′) with the packaging plasmids psPAX2 and pM2D.G; and used to transduce U2OS cells, followed by selection with puromycin (2.5 μg/ml).

### Generation of TP53 knockout MDA-MB-231 clones

CRISPR-mediated *TP53* knockout MDA-MB-231 cells were generated using an all-in-one pD1401-AD plasmid expressing the Cas9 nickase (Cas9-D10A), GFP and the 2 gRNAs targeting the *TP53* exon that was common to all p53 mRNA isoforms (Supplemental Figure 5). MDA-MB-231 cells were transfected with 1 μg of plasmid by Nucleofection using Amaxa^®^ Nucleofector^®^ Kit V (Catalog # VCA-1003). After 48 h, transfected cells were GFP sorted and seeded at one cell per well in 96-well plates containing DMEM. Single colonies were expanded and protein was extracted using RIPA buffer. Knockout clones were genotyped by Sanger sequencing and p53 loss was confirmed by immunoblotting using an Anti-p53 antibody (DO1, Santa Cruz catalog number sc-126). gRNAs sequences targeting TP53: gRNA1- GATGGCCATGGCGCGGACGC; gRNA2- GCAGTCACAGCACATGACGG.

### Preparation of RNA and qRT-PCR

Total RNA was extracted from cells using Trizol (Life Technologies). The quantification of the extracted RNA was done using a NanoDrop 2000c Spectrophotometer (Thermo Scientific). For quantitative reverse transcription-PCR (RT-qPCR) analysis, 500 ng of total RNA was reverse transcribed using the iScript RT kit (Bio-Rad), and qPCR was performed using SYBR green (Bio-Rad) as directed by manufacturer. The glyceraldehyde-3-phosphate dehydrogenase (GAPDH) and β-Actin housekeeper genes were used as internal control. The qPCR for each RNA sample was performed in triplicate. No template control was used to rule out cross contamination of reagents and a RT minus control was used to rule out genomic DNA contamination. Statistical significance of gene expression changes in untreated versus treatment groups was confirmed using a two-tailed paired Student-t test and is presented in the figures as *p < 0.05; ^#^*p* < 0.01; **p < 0.005; ^##^*p* < 0.001; or n. s., non-significant. The oligonucleotide sequences of the primers used for qRT-PCR are as following: RECQ1 #1 (Forward: 5′-CAATGGCTGGAAAGGAGGTA-3′; Reverse: 5′-AATGGGCAAATGACGAGTGT-3′), RECQ1 #2 (Forward: 5′-TGAAGCAGGCAGAGGAACTG-3′; Reverse: 5′-AGCCACAACACCTGCTACTC-3′); GAPDH (Forward: 5′-AATCCCATCACCATCTTCCA-3′; Reverse: 5′-TGGACTCCACGACGTACTCA-3′), β-actin (Forward: 5′-ACCAACTGGGACGAT ATGGAGAAGA-3′; Reverse: 5′-TACGACCAGAGGCATACAGGGACAA-3′), p21 (Forward: 5′-GACTCTCAGGGTCGAAAACG3′; Reverse: 5′-GGAT TAGGGCTTCCTCTTGG-3′), WRN (Forward: 5′-AATC TACTGAGCATTTATCTCCCA-3′; Reverse: 5′-GAGTT GGTTCTACCGTGCCA-3′), BLM (Forward: 5′-GAGT CTGCGTGCGAGGATTA-3′; Reverse: 5′-AGTGTTC TGGCTGAGTGACG-3′), RECQ4 (Forward: 5′-TCACAG TGAGGTCCCAGATT-3′; Reverse: 5′-CTGACTTCTT GGAAGGCTGA-3′), RECQ5 (Forward: 5′-GCTCAGG AAGACGGGAGAAG-3′; Reverse: 5′-AGAACAGCTT GGAGAACGGG-3′). Following two primer pairs were used for mouse: RECQ1 (Forward: 5′-GCTCTTGGCAT CTTGAAGCG-3′; Reverse: 5′-CTTGAGGGCTTTTGCC GAAC-3′) and GAPDH (Forward: 5′-CGTGTTCCTACCC CCAATGT-3′; Reverse: 5′-GTGTAGCCCAAGATGCCC TT-3′).

### Western blotting

Whole-cell lysates were prepared by using radioimmunoprecipitation assay (RIPA) buffer containing protease inhibitor cocktail (Roche), and protein was quantified using DC protein assay kit (Bio-Rad). Ten microgram of total protein per lane was used for immunoblotting. The following primary antibodies were used: anti-RECQ1 (Bethyl lab; at 1:1000 dilution); anti-p53 (Santa Cruz Biotech, 1:2,500 dilution), anti-p21 (Santa Cruz Biotech, 1:500 dilution), anti-GAPDH and anti-γH2AX (both from Cell Signaling, 1:1000 dilution).

### Chromatin immunoprecipitation assay

Cells grown at a density of 1 × 10^7^ per 15 cm diameter dish, untreated or after MMS treatment (1 mM, 8h), were used for ChIP experiments as described previously [68]. Following phenol/chloroform extraction and ethanol precipitation, sheared DNA fragments served as template in qPCR analysis. qPCR was performed using Taq Universal SYBR Green Supermix (Bio-Rad) with technical triplicates and threshold cycle numbers (C_t_) were determined with an iQ5 thermal cycler (Bio-Rad). Fold enrichment of the targeted genomic sequences were calculated over IgG as: fold enrichment = 2^-(CtIP− CtIgG)^, where Ct_IP_ and Ct_IgG_ are mean threshold cycles of PCR done in triplicates on DNA samples immunoprecipitated with anti-p53 (DO-1, Santa Cruz Biotech) or RNA POL II (CTD4H8 phospho-S5 [4H8], Abcam) antibody and control IgG, respectively. All qPCR reactions were also checked by melt curve analyses and agarose gel electrophoresis to confirm the presence of a single specific product. Statistical significance of enrichment in ChIP in untreated versus treatment groups was confirmed using a two-tailed paired Student-t test for each genomic sequence and is presented in the figures as *p < 0.05; ^#^*p* < 0.01; **p < 0.005; ^##^*p* < 0.001; or n. s., non-significant. Following primers were used: primers for -819/+21 region of *RECQ1* promoter: #1 (Forward: 5′-TGCTTCACAGTAGCGGAAGG-3′; Reverse: 5′-ATGTTGGAGGAAACGCCACT-3′); #2 (Forward: 5′-CCGGTCTTCTGATCTCCCCA-3′, Reverse: 5′-TTAATAACGCCCGCCCTTCC-3′); #3 (Forward: 5′-T GCCTCTAAATGCAGGTGGC-3′; Reverse: 5′- GCA GGTCTGTCACTCAGCAG-3′), primer to the p21 promoter containing p53 RE as positive control (Forward: 5′-CTGGACTGGGCACTCTTGTC-3′; Reverse: 5′- CTC CTACCATCCCCTTCCTC-3′), and primers to GAPDH as a p53 irrelevant and non-promoter region (Forward: 5′-TACTAGCGGTTTTACGGGCG-3′; Reverse: 5′-TCGCTCTCTGCTCCTCCTGTTCGA-3′).

### RECQ1 promoter construct

The 5′-region from −819 bp to +21 bp, relative to the transcription start site of RECQ1 (Gene ID 5965) was cloned by PCR of genomic DNA (forward primer, 5′-AGCCAGGGACTTGTAGAGGAC-3′; reverse primer, 5′-TGGCGAAACCTGCTTCCAA-3′). The PCR product was cloned into the pGL3-Basic luciferase vector (Promega) using standard techniques. All constructs were sequence-verified.

### Luciferase reporter assay

HCT116 cells, p53-proficient or p53-deficient, at 60% confluence in 6-well plates were co-transfected with 500 ng pGL3-Basic luciferase reporter constructs containing a sequence derived from the identified p53-binding site upstream of the *RECQ1* gene, (PGL3_RECQ1), wt p53 binding sites (PGL3_p53RE; a gift from Bert Vogelstein (Addgene plasmid # 16442)[41], or empty vector and 50 ng pRL-TK Renilla reporter (Promega) by Lipofectamine 2000 as described by the manufacture's protocol. Cells were lysed 36 h post-transfection and luciferase activity was measured using the Dual-Luciferase Reporter Assay System (Promega) according to protocol. Firefly luciferase activities were normalized with Renilla luciferase activities to obtain the relative luciferase activity. Luciferase activity for pGL3_RECQ1 was comparable to that of pGL3_p53RE. Results are presented as normalized relative luciferase activity compared to p53-proficient HCT116 cells.

### Survival assay

Cells, seeded at a density of 3 × 10^3^ cells per well in 96 well plates, were either untreated or treated with DNA damaging agents in complete DMEM medium as indicated. Cell proliferation was evaluated each day by trypan blue exclusion assay and counting the viable cells using an automated cell counter (Bio-Rad TC10). Cell proliferation experiments were done in triplicates in three independent experiments.

### Comet assay

The neutral comet assays were performed using a Comet Assay kit (4250-050-ESK, Trevigen) according to the manufacturer's recommendations. Cells untreated or treated with doxorubicin (1 μM) or MMS (1 mM) for 4 h were trypsinized and embedded on a microscope slide in agarose. Slides were incubated for 30 min at 4°C in a CO_2_ incubator. Cells on slides were lysed for 1 h at 4°C and the slides were then incubated in the dark for 30 min in cold neutral electrophoresis buffer prior to electrophoresis at 25 V for 25 min. After immersion of slides for 30 min each in DNA precipitation solution and 70% ethanol, DNA was visualized using SYBR Green I fluorescent staining. Fifty cells per sample were documented in each case. Data was analyzed using an open-source software tool, OpenComet. Results are presented as normalized % tail moment compared to untreated control knockdown cells.

### *RECQ1* gene expression in human breast cancers

We initially investigated *RECQ1* gene expression in the Breast Invasive Carcinoma TCGA cohort [54] using the publicly available cBioPortal for Cancer Genomics [69]. We then validated predictive significance of *RECQ1* in a large cohort of 3951 breast cancers [55]. Relapse free survival was defined as the number of months from diagnosis to the occurrence of local recurrence, local lymph node (LN) relapse or distant metastasis (DM) relapse.

## SUPPLEMENTARY MATERIALS FIGURES AND TABLES



## Abbreviations

All abbreviations have been defined within the manuscript.
