# Supplementary material for: RECQ1 expression is upregulated in response to DNA damage and in a p53-dependent manner
Source: Oncotarget. 2017 May 27;8(44):75924–42. doi: 10.18632/oncotarget.18237 (PMC5652675; doi:10.18632/oncotarget.18237)
Supplement: Supplementary file 1 [file oncotarget-08-75924-s001.pdf]

RECQ1 expression is upregulated in response to DNA damage and in a p53-dependent manner

Supplementary Materials

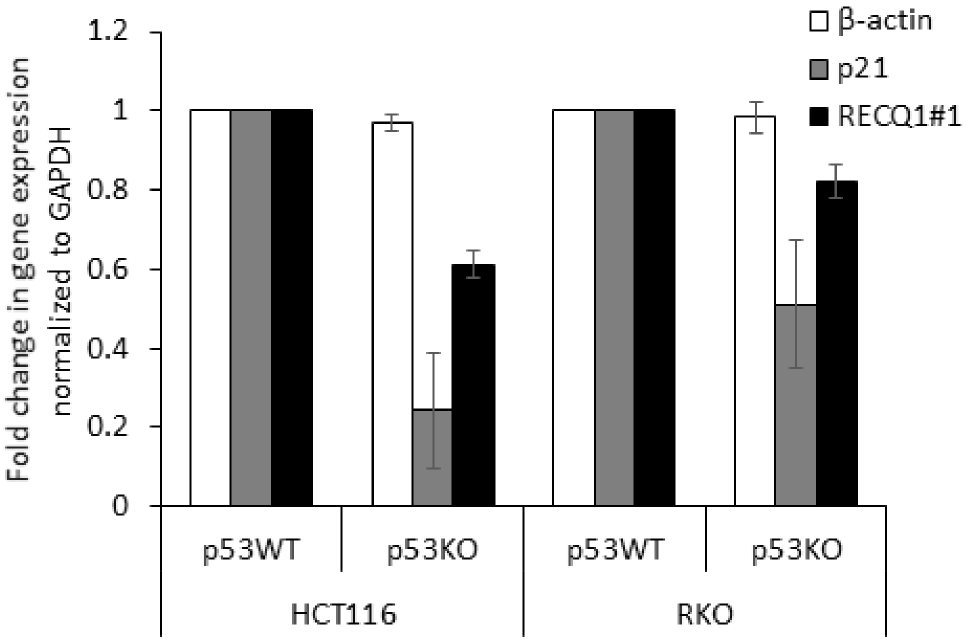

Supplementary Figure 1: Basal expression of RECQ1 in p53 wild-type and p53 knockout cells. RECQ1 and p21 expression was measured; β-actin served as additional housekeeping control. Fold-change in expression compared to untreated and normalized to GAPDH is shown. Values are average of three independent experiments and standard deviation is indicated by error bars.

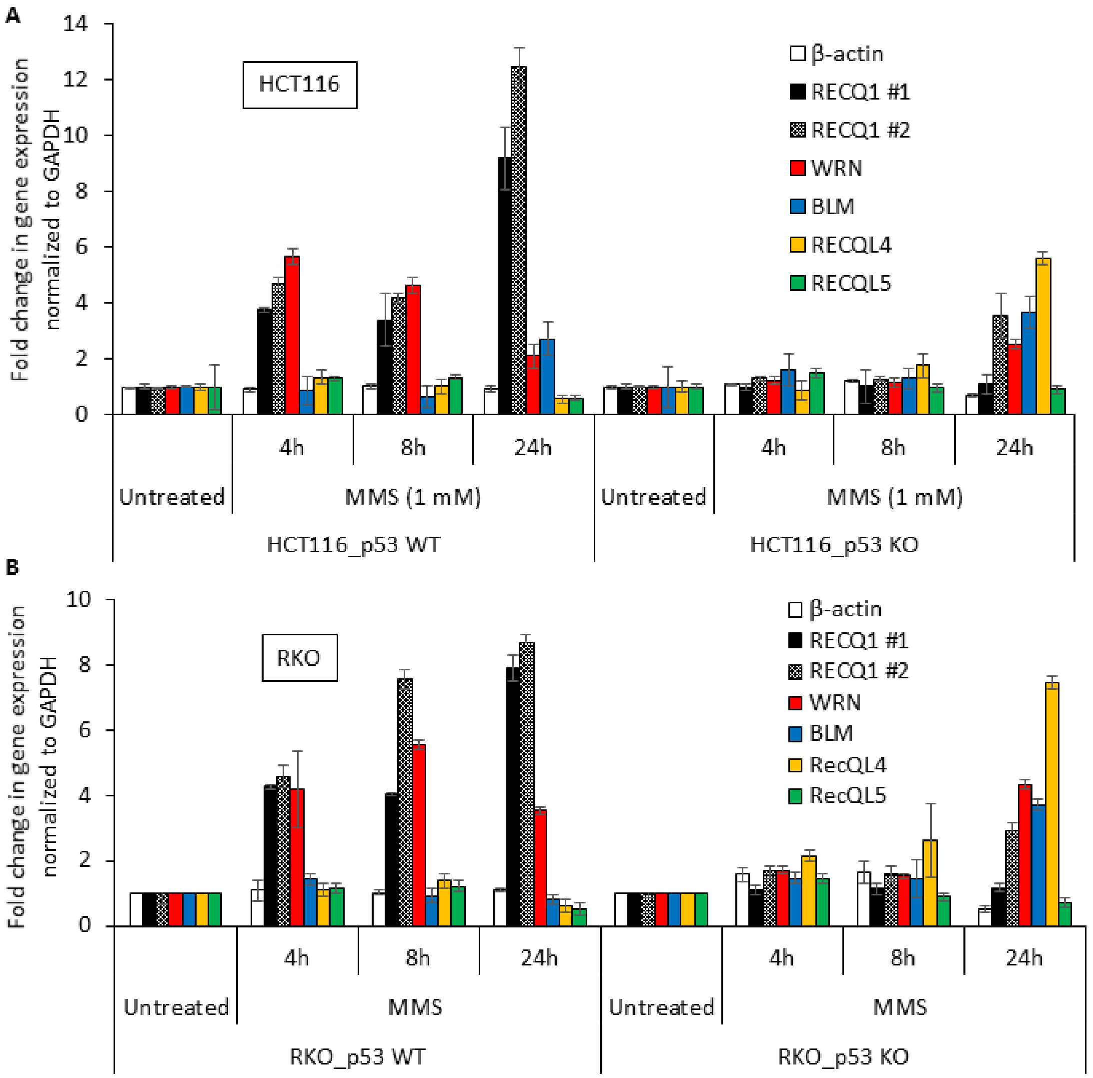

Supplementary Figure 2: Comparative analysis of MMS-triggered changes in gene expression for five RecQ members in isogenic p53 wild-type and p53 knockout cells. RNA was isolated from HCT116 (**A**) or RKO (**B**) cells that were untreated or treated with 1 mM MMS for indicated time periods. Fold change in mRNA expression of RECQ1, WRN, BLM, RECQ4 and RECQ5 compared to untreated and normalized to GAPDH is shown. β-actin served as additional housekeeping control. Values are average of three independent experiments and standard deviation is indicated by error bars.

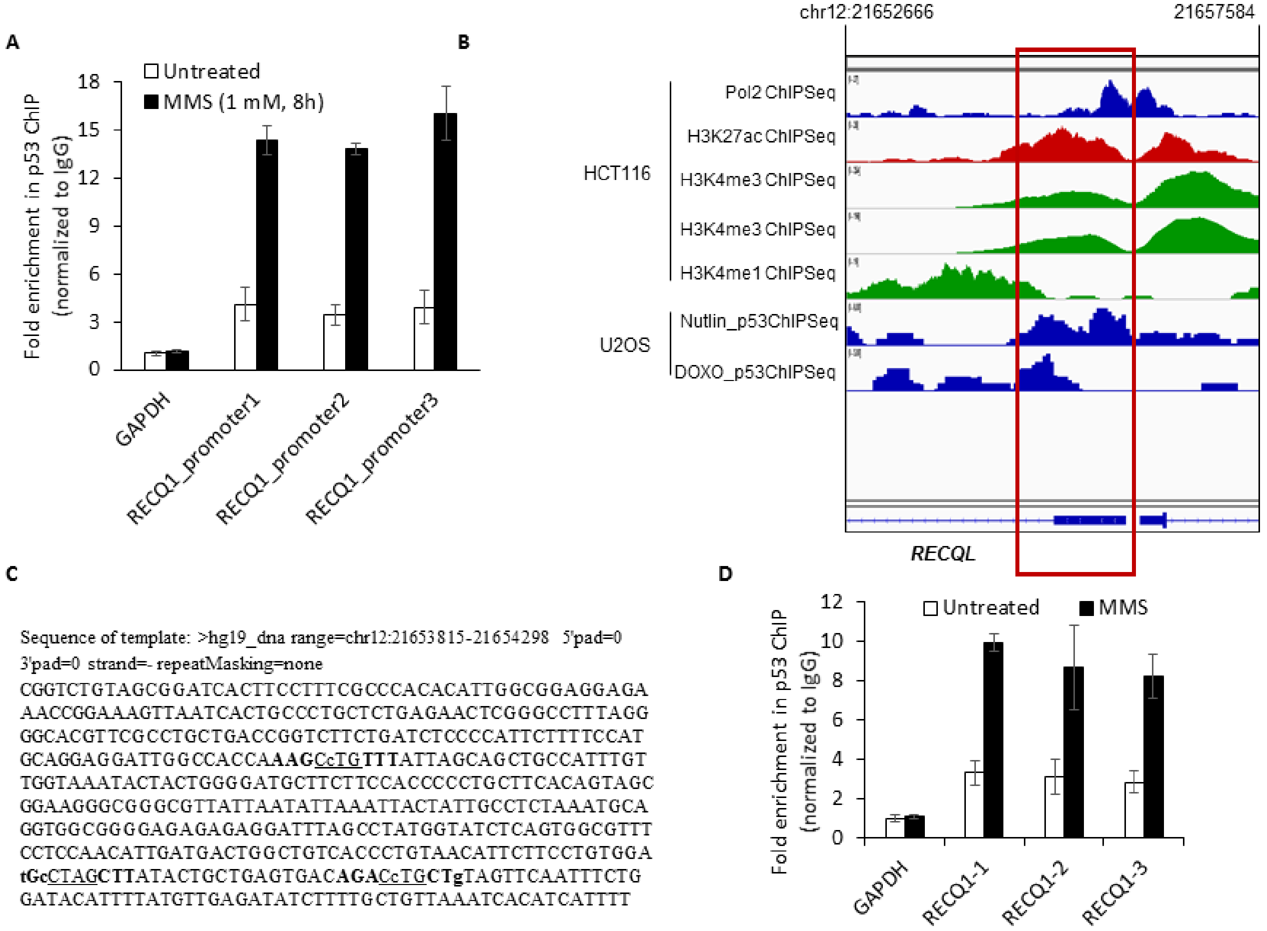

Supplementary Figure 3: p53 binding on RECQ1 promoter and gene in U2OS cells after MMS treatment. (**A**) Enrichment of p53 to RECQ1 promoter in U2OS cells untreated or treated with MMS (1 mM, 8 h) as determined by ChIP-qPCR using a p53-specific antibody. ChIP-qPCR of immunoprecipitated DNA with three probes specific for RECQ1 promoter sequence containing predicted p53 binding sites (#1, #2, and #3 as used for HCT116 cells). Fold enrichment in p53 ChIP over rabbit IgG was determined and is shown for each primer pair for the ChIP. Results are expressed as means ± SEM for at least three independent experiments. (**B**) Analysis of p53 ChIP-seq data from Menendez et al [47] in doxorubicin treated U2OS cells indicates p53 binding site in RECQ1 exon 1 and maybe intron 1. (**C**) Genomic sequence corresponding to RECQ1 exon 1 with three putative non-canonical p53-binding sites shown in bold. (**D**) Enrichment of RECQ1 gene sequence corresponding to exon 1 with three putative non-canonical p53-binding sites in p53-ChIP from U2OS cells, untreated or MMS treated (1 mM, 6 h). RECQ1-1: Amplicon size = 120 bp; FP1: TGCTTCACAGTAGCGGAAGG; RP1: ATGTTGGAGGAAACGCCACT; RECQ1-2: Amplicon size = 148 bp; FP2: CCGGTCTTCTGATCTCCCCA; RP2: TTAATAACGCCCGCCCTTCC; RECQ1-3: Amplicon size = 144 bp; FP3: TGCCTCTAAATGCAGGTGGC; RP3: GCAGGTCTGTCACTCAGCAG.

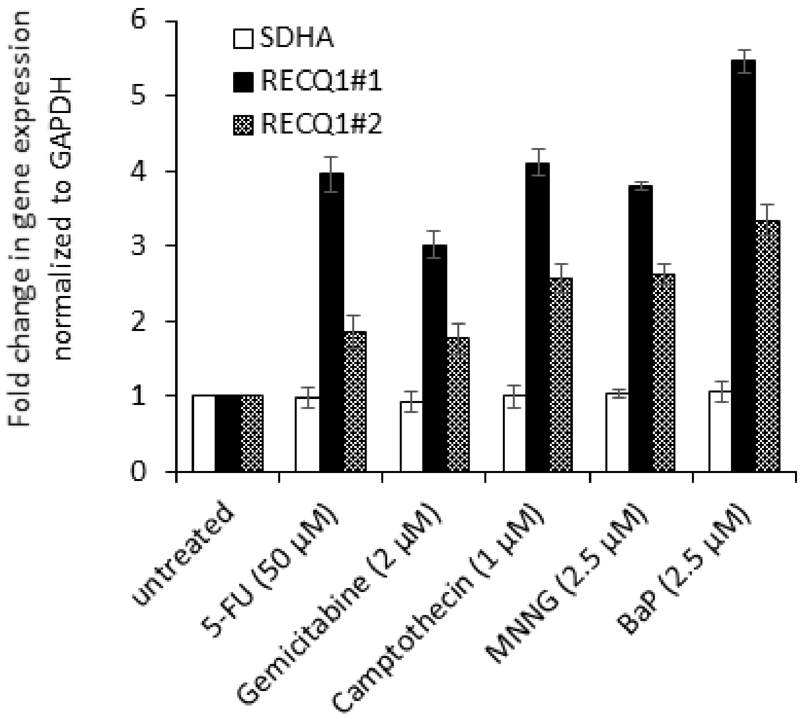

Supplementary Figure 4: RECQ1 expression is upregulated following treatment with a variety genotoxic agents. RNA was isolated from U2OS cells that were untreated or treated for 6 h with indicated drugs at concentrations as specified. RECQ1 mRNA expression was measured using two independent primer sets; fold-change in expression compared to untreated and normalized to GAPDH is shown. SDHA served as additional housekeeping control. Values are average of three independent experiments and standard deviation is indicated by error bars.

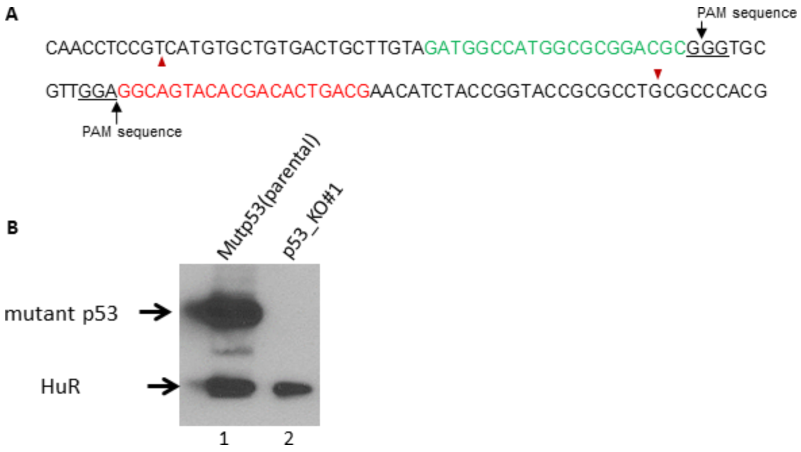

Supplementary Figure 5: Generation of TP53 knockout MDA-MB-231 clones. (**A**) DNA sequence representing the TP53 exon common among different isoforms and targeted for CRISPR. Red and green font represents gRNA sequences targeting the top and bottom alleles of TP53. PAM sequences are underlined and Cas9 cleavage site is shown in red arrows. (**B**) Screening of CRISPR clones for p53 expression by Western Blot. Protein level of HuR in p53-CRISPR clones was checked as an unrelated control. Lane 1 contains parental MDA-MB-231 cells harboring mutant p53. The p53 protein was not detected in KO#1 (lane 2).

A

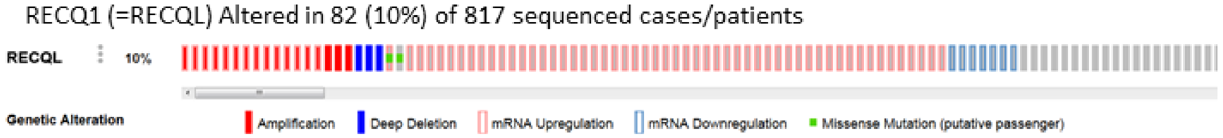

B

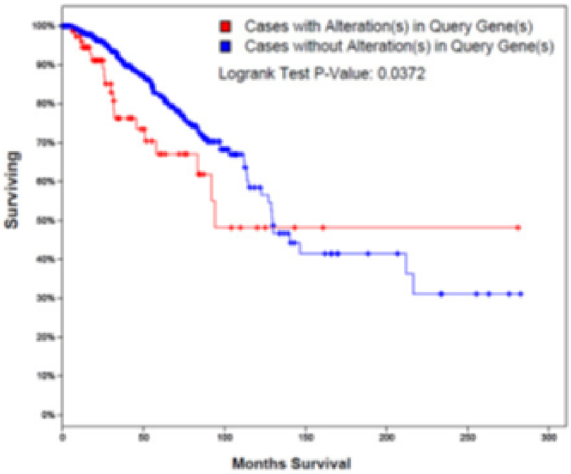

Supplementary Figure 6: Clinical correlation of RECQ1 expression. (**A**) Oncoprint of RECQ1 genetic alterations in TCGA Breast Invasive Carcinoma [54]. (**B**) High RECQ1 expression correlates with significantly poor overall survival of breast cancer patients in this dataset.
